# Positive modulation of sigma-1 receptor: a new weapon to mitigate disease progression in amyotrophic lateral sclerosis

**DOI:** 10.1186/s40035-025-00527-z

**Published:** 2025-12-15

**Authors:** Julien Le Friec, Hugo Mourier, Simon Couly, Nicolas Cubedo, Kevin Dubois, Johann Meunier, Benjamin Delprat, Arnaud De Zordo-Banliat, Tahar Ayad, David Virieux, Tsung-Ping Su, Christelle Lasbleiz, Tangui Maurice, Jean-Charles Liévens

**Affiliations:** 1 MMDN, Univ Montpellier, EPHE, INSERM, Montpellier, France; 2 Cellular Pathobiology Section, Integrative Neuroscience Research Branch, Intramural Research Program, National Institute On Drug Abuse, NIH, 333 Cassell Drive, Baltimore, MD 21224 USA; 3 ICGM, Univ Montpellier, ENSCM, CNRS, Montpellier, 34090 France; 4 PSL Research University, Paris, France

**Keywords:** Amyotrophic lateral sclerosis, Sigma-1 receptor, Tar-DNA binding protein 43 kDa, C9orf72, NRF2 signalling

## Abstract

**Background:**

Amyotrophic lateral sclerosis (ALS) is characterised by degeneration of motor neurons, leading to muscle weakness and progressive paralysis. Currently, no treatment is available to halt or reverse the progression of the disease. Oxidative stress, mitochondrial dysfunction, accumulation of unfolded proteins and inflammation are interconnected key actors involved in ALS. A potent therapeutic strategy would be to find molecules that break this vicious circle leading to neuronal dysfunction and death. Targeting sigma-1 receptor (S1R) could meet this objective, as this chaperone protein modulates many cell survival mechanisms. So far, the impact of S1R activation in ALS has been studied using specific agonists and mostly on the *SOD1* mutation that represents only 2% of patients. In the present study, the impact of two different S1R activators, the reference agonist PRE-084 and the positive modulator OZP002, was compared on two key ALS genes: *TDP43* and *C9orf72*.

**Methods:**

The dissociation of S1R from Binding immunoglobulin Protein (BiP) was determined using ELISA. OZP002 toxicity was compared to PRE-084 on zebrafish larvae with increasing concentrations. The efficacy of OZP002 and PRE-084 was evaluated on the locomotor escape response of zebrafish expressing mutant TDP43 or one C9orf72 toxic dipeptide. Their effects on NRF2 target gene expression were studied by qPCR. The beneficial effect was further examined on the locomotor performances of TDP43^A315T^ mice using rotarod and beam walking tests. We also performed analysis on motor neuron loss and glial reactivity.

**Results:**

OZP002 is a positive modulator of S1R, that increases the dissociation of the S1R-BiP complex induced by orthosteric agonists. S1R activation by both OZP002 and PRE-084 restored the locomotor response of ALS zebrafish expressing either TDP43 or one C9orf72 toxic dipeptide. The neuroprotection was due at least in part to the NRF2 cascade stimulation but not with a direct interaction. More importantly, OZP002 and PRE-084 prevented locomotor defects and degeneration of spinal motor neurons in TDP43^A315T^ transgenic mice. Astroglial and microglial reactivities were also reduced by both activators.

**Conclusions:**

We here emphasize the therapeutic value of S1R activation in mitigating ALS pathology. Additionally, we show that the positive modulators pave the way for the development of new S1R-activating compounds for ALS treatment.

**Supplementary Information:**

The online version contains supplementary material available at 10.1186/s40035-025-00527-z.

## Background

Amyotrophic lateral sclerosis (ALS) continues to be a devastating and fatal neurodegenerative disease despite extensive research. Disease progression is usually fast with most of the patients displaying at early-stage muscle atrophy, paralysis, respiratory failure and dying within 2–5 years after disease onset. Up to 50% of patients also develop cognitive impairments such as frontotemporal dementia. Among the diagnosed ALS patients, 10% are familial cases with a pure genetic inheritance and, the remaining 90% are sporadic cases with a combination of both genetic and environmental factors [1]. To date, more than 40 genes are causative for ALS, with most mutations following an autosomal-dominant inheritance [2]. The most common ALS-linked genes encode the copper/zinc superoxide dismutase 1 (SOD1), the chromosome 9 open reading frame 72 (C9orf72), TAR DNA-binding protein of 43 kDa (TDP43) or fused in sarcoma (FUS) proteins. Mutations in *C9orf72* are by far the most frequent causes of ALS, contributing to 40%–50% of familial and 5%–10% of sporadic ALS in Europe and North America [3, 4]. Importantly, a histological hallmark in the majority of ALS cases is the mislocalisation of TDP43 from the nucleus in favour of cytoplasm [5], suggesting that the ALS-causing mechanisms may converge on this central pathological pathway. *C9orf72* mutation and TDP43 mislocalisation are also found in patients with frontotemporal lobar degeneration (FTLD), suggesting that ALS and FTLD represent two extremes of a disease spectrum. To date, only two medicines have been approved by both the US Food and Drug Administration and the European Medicines Agency: Riluzole which prolongs life by only a few months with limited effects on motor deficits [6] and Tofersen, an antisense oligonucleotide only dedicated to the 1%–2% of ALS cases with mutations in *SOD1* [7, 8]. Thus, most patients have no access to a treatment to halt or reverse the disease. Oxidative stress is a common phenomenon in neurodegenerative diseases including ALS. It results from an imbalance between antioxidant defence and reactive oxygen species production. Oxidative stress represents a critical component in a vicious circle, supporting mitochondrial dysfunction, neuroinflammation, endoplasmic reticulum (ER) stress and vice versa. Identifying strategies to break out of this deleterious loop may pave the way for delaying neuronal dysfunction and loss.

S1R has recently drawn increasing attention in ALS due to its potential therapeutic impact. Furthermore, the E102Q mutation in S1R was reported to cause ALS with a juvenile onset [9]. S1R is a molecular chaperone mainly located in the ER, more particularly at the mitochondria-associated ER membrane. It amplifies pre-existing cellular mechanisms of neuroprotection such as calcium-mediated mitochondrial functions, neurotrophic support, ER stress response, cellular redox homeostasis and neuroinflammation [10–12]. S1R is also a receptor with activity modulated by endogenous agents such as the neurosteroids, dehydroepiandrosterone (DHEA) and pregnenolone for activation or progesterone for inhibition. Of interest, multiple synthetic small molecules have been designed to target S1R for clinical purpose.

S1R activation by agonists may confer neuroprotection in ALS. However, this view is mainly supported by studies using models bearing *SOD1* mutation [13–17]. Further analyses are required to demonstrate that S1R is a more general target to alleviate ALS pathology. Only recently, we found that the reference S1R agonist, PRE-084, also alleviated the motor phenotype of zebrafish larvae expressing mutant TDP43 [18]. Another approach for drug design is based not on the main orthosteric site but on secondary sites that modulate the impact of endogenous agonists. Targeting such sites may provide enhanced selectivity, limited adverse effects and reduced toxicity. Such positive modulators have been identified for S1R [19]. To our knowledge, no S1R positive modulator has been evaluated in the ALS context. In the present study, we addressed the efficacy of the positive S1R modulator OZP002 [20] in ALS models. OZP002 was compared to the reference agonist PRE-084 in zebrafish larvae expressing either mutant TDP43 or one C9orf72 toxic dipeptide. For the first time, the impact of S1R activation was also examined on the locomotor deficits, motor neuron loss and glial reactivity in a murine TDP43 model.

## Methods

### Drugs

2-(4-Morpholinethyl)-1-phenylcyclohexanecarboxylate hydrochloride (PRE-084; P2607) and DHEA (252805) were purchased from Merck (Lyon, France). 4-Methoxy-3-(2-phenylethoxy)-N,N-dipropyl-benzeneethanamine hydrochloride (NE-100; 3133) was purchased from Tebubio (Le Perray-en-Yvelines, France). 2-(3-Chlorophenyl)-3,3,5,5-tetramethyl-2-oxo-[1,4,2]-oxazaphosphinane (OZP002) was synthesized as previously described [20]. Stock solutions of OZP002, PRE-084 and NE-100 were prepared in water at 10 mM for zebrafish treatment or 1 mg/mL for mouse treatment. DHEA was solubilized in polyethylene glycol 400 (PEG-400) at 10 mM. Drugs were diluted either in fish water or in 0.9% saline solution (vehicle) for intraperitoneal injection in mice. The final concentration of PEG-400 in water was adjusted to 0.15% for DHEA treatment or corresponding control.

## S1R/BiP dissociation assay

The assay was performed as described by Hayashi and Su [21] with modifications. S1R-GFP-expressing CHO cells were maintained in DMEM/GlutaMAX culture medium (ThermoFisher, Asnière-Sur-Seine, France) supplemented with 10% foetal bovine serum (ThermoFisher). Cells were plated in 12-well plates, and then treated with compounds dissolved in culture medium for 30 min at 37 °C. Cells were rinsed with phosphate buffer saline 1× (PBS), harvested, centrifuged for 5 min at 12000 *g* and then re-suspended in 1× PBS. Protein cross-linking was performed using 50 μg/mL of dithiobis(succinimidyl-propionate) (ThermoFisher) for 30 min at 4 °C, and reaction was stopped by Tris/HCl 50 mM (pH 8.8). Cells were lysed in RIPA buffer (50 mM Tris pH 7.4, 150 mM NaCl, 1% Triton X-100, 0.3% sodium deoxycholate, and 0.1% sodium dodecyl-sulphate) containing a protease inhibitor cocktail Mini cOmplete (11836153001; Merck) for 15 min on ice. After centrifugation at 16,000 *g* for 1 min, the supernatant was incubated with ChromoTek GFP-Trap agarose (Proteintech, Manchester, United Kingdom) overnight at 4 °C. Cell lysates were incubated with Sepharose Protein-A (Invitrogen-Thermo Fisher Scientific, Courtaboeuf, France) for 90 min at 4 °C. After centrifugation at 12,000 *g* for 1 min, the pellets were suspended in RIPA buffer for 30 min. After a final centrifugation at 12,000 *g* for 1 min, pellets were suspended in PBS and stored at 4 °C. Samples were processed using the Heat Shock 70 kDa Protein 5 ELISA assay (#CL-SEC343Mu, Euromedex, France) following the manufacturer’s protocol.

## Zebrafish housing and injection

Zebrafish (*Danio rerio*) Tübingen strain was bred and maintained in ZEFIX fish facility (MMDN, Montpellier, France). Adult fishes were maintained at 28 °C, pH 7 with 14 h/10 h light/dark cycle in a stand-alone housing unit ZebTEC (Tecniplast, Lyon, France) and fed twice a day with pellet of Gemma Micro 500 (Skretting, France). All experimental procedures on zebrafish were performed in the fish facility in accordance with the 2010/63/EU Directive and the ARRIVE guidelines [22].

Transcripts encoding human wild-type TDP43 (TDP43^WT^), mutant TDP43 (TDP43^G348C^), 100 glycine-arginine repeats (GR^x100^), or the zebrafish nuclear factor erythroid-2 related factor 2a (nrf2a) isoform were generated from the previously reported plasmids [23–25]. The mCherry coding sequence was used to check the correct injection of embryos. Plasmids were linearized using Not1 restriction enzyme, then transcribed using the SP6 or T7 polymerase from mMESSAGE Machine kit (Invitrogen-Thermo Fisher Scientific, Courtaboeuf, France) and purified according to the manufacturer’s instructions. Antisense morpholino (5’-CATTTCAATCTCCATCATGTCTCAG-3’) to downregulate *nrf2a* has been described previously [26] (Gene Tools, Philomath, Oregon). Control standard morpholino (5’-CCTCTTACCTCAGTTACAATTTATA-3’) (Gene Tools) was used to assess off-target effects.

A volume of 1 nL was injected into each embryo at the one-two cell stage with the following concentrations, depending on the experimental conditions: *TDP43*^*G348C*^ mRNA (40 ng/μL); *GR*^*x100*^ mRNA (20 ng/µL); *STOP-GR*^*x100*^ mRNA (20 ng/µL); *nrf2a* mRNA (20 ng/μL), *nrf2a* morpholino (0.4 mM) or standard control morpholino (0.4 mM). At 1-day post-fertilisation (dpf), malformed larvae were discarded and only larvae with a suitable expression level of red fluorescent mCherry were sorted using a fluorescent stereomicroscope (Olympus, MVX10).

## Fish acute toxicity assay

Larvae at 4 dpf were plated into 10-cm petri dishes (24 larvae per petri dish) containing increasing doses of PRE-084 or OZP002 (5 to 104 µM). After an incubation at 28 °C for 24 h, larvae were observed with a binocular (Motic SMZ-171; Motic, Wetzlar, Germany) to determine if they were alive, dead (necrotic body or lack of heartbeat for more than 30 s) or exhibiting malformations (pericardiac oedema, kyphosis, lordosis or prominent jaws).

To evaluate if compounds affect the locomotor behaviour, 5-dpf larvae were transferred to 48-well plates and acclimatized for 60 min in an incubator at 28 °C. Then, the plates were placed into a Zebrabox recording chamber build by ViewPoint from ZEFIX platform (MMDN, Montpellier, France). Free locomotor activity was recorded in light environment at 28 °C for 60 min and then analysed using Zebralab software. Statistical analysis was performed by including at least 20 larvae per test condition.

## Touch-escape test

The touch-escape test quantifies the locomotor activity of zebrafish larvae following a mechanical stimulation [18]. Treatments with S1R activators were performed at 4 dpf for 24 h. At 5 dpf, individual zebrafish larvae were transferred to the extremity of a longitudinal tank (L 17.5 × W 0.5 × H 1 cm^3^) filled with 2.6 mL of zebrafish water and equipped with a graduated scale. After an acclimatization period of 30 s, a tactile stimulation was performed with a tip on the tail fin. The distance swam in 5 s was measured. The larvae were then placed again at the extremity, and two other trials were performed after a rest period of 30 s between each session. The experimenter was kept blind during the test. Data from the 3 consecutive assays were then averaged for each zebrafish.

## Quantitative real-time PCR (qPCR)

Total RNA was extracted from whole zebrafish larvae at 5 dpf (*n* = 8 larvae/tube) using the NucleoSpin RNA kit (740955.55; Macherey Nagel, Düren, Germany), following the manufacturer’s instructions. RNA concentration and quality were evaluated using a NanoDrop One spectrophotometer (Thermo Fisher Scientific, Les Ulis, France). Reverse transcription was performed using M-MLV Reverse Transcriptase (M170V; Promega, Charbonnières-les-Bains, France) following the manufacturer’s instructions. Sybr No-Rox Mix (Bio98050; Sensifast, Bioline, Ozyme, Saint-Cyr-l’École, France) and cDNAs were distributed on reaction plates (μltraAmp PCR plate, Sorenson BioScience, Murray) using an Echo 525 acoustic liquid handler (Labcyte, Sunnyvale, CA). Real-time PCR amplification was performed with 45 cycles (10 s at 95 °C, 10 s at 60 °C, and 10 s at 72 °C) using a LightCycler 480 (Roche, Boulogne-Billancourt, France). Fold changes in gene expression were analysed using the 2^−ΔΔCT^ method [27]. Specific primers for amplification of genes are listed in Table S1, and expression levels were normalized to the housekeeping gene ETS-related transcription factor, EF1α.

## Immunoprecipitation

S1R knocked-out (S1R-KO) N2a cells generated with CRISPR-Cas9 as previously described with a stabilised transfection allowing the expression of YFP or S1R-YFP were seeded in 10-cm dishes at a concentration of 2 × 10^5^ cells/dish [28]. After 48 h, cells were washed with 1 mL 1× PBS and harvested with 500 μL of PBS lysed using CHAPS lysis buffer (50 mM Tris pH 7.4, 150 mM NaCl, 0.5% CHAPS) and supplemented with EDTA-free protease inhibitor cocktail tablets (Mini COmplete; 11836170001; Roche Diagnostics, Indianapolis, IN) on ice for 30 min. Protein concentrations of cell lysates were determined using BCA protein assay kit (23225; Thermo Fisher Scientific, Waltham, MA). 1 μg of anti-GFP rabbit or mouse antibody (Table S2) or rabbit or mouse normal IgG (Table S2) was added to 5 μL of Dynabeads Protein G (100003D; Invitrogen, Carlsbad, CA), then mixed and incubated at room temperature for 15 min. Then, 300 μg of cell lysate were added to the antibody-beads mixture and mixed overnight at 4 °C. Beads were washed with 500 μL of CHAPS lysis buffer three times, then beads were resuspended in 30 μL of 2× Laemmli sample buffer and heated at 70 °C for 15 min. These protein samples were separated by 14% SDS-PAGE containing 2% 2,2,2-Trichloroethanol to measure the concentration of protein in each lane and transferred onto a polyvinylidene difluoride membrane. After incubation with 2% BSA in Tris Buffer Solution + 0.1% Tween 20 (TBS-T) for 1 h, membranes were incubated with primary antibodies for 1 h at room temperature. The following antibodies were used: anti-BiP, anti-Kelch-like ECH-associated protein 1 (Keap1), anti-NRF2 and anti-S1R (Table S2). Membranes were washed 3 times with TBS-T for 10 min followed by secondary antibody for 1 h at room temperature. Blots were washed 3 times for 10 min with TBS-T and developed by using the Li-Cor odyssey CLx (LICORbio™, Lincoln, NE). Band intensity was analysed by Image Studio Lite (LiCor 5.2.5) according to the manufacturer’s manual.

## Mouse colony maintenance and treatments

All animal procedures were conducted in strict adherence to the European Union Directive 2010/63 and the ARRIVE guidelines [22], and were approved by the National Ethic Committee (Paris) (APAFIS #33337–2021093015356901 v6). Mice were housed in a temperature- and humidity-controlled animal facility on a 12 h/12 h light/dark cycle (lights on at 7:00 h) at the university of Montpellier (registration number D34-172-23). They were kept in groups with access to food and water ad libitum. All behavioural experiments were performed during the light cycle.

Hemizygous transgenic mice expressing the human TDP43^A315T^ transgene under the regulation of the prion promoter were purchased from the Jackson Laboratory (strain 010700, Jackson Laboratory, Bar Harbor, ME). To maintain the colony, TDP43^A315T^ males were mated with pure C57BL/6 J mouse females (Janvier Labs, Le Genest-Saint-Isle, France). Pups were weaned at age 21 days and mice were monitored at least 3 times a week to avoid loss of the colony. Due to gastrointestinal complications prior to the development of full neurological ALS symptoms [29], mice were fed with a gel-based diet**,** DietGel 76A (ClearH_2_O, Westbroock, ME) [30] and housed in cages without wood fibers or cardboard houses. PCR-based genotyping of ear DNA was used to identify the transgenic and littermate control mice. Genotyping was performed as described by the Jackson Laboratory.

Intraperitoneal injection of 0.9% vehicle saline solution, OZP002 (0.7 mg/kg) or PRE-084 (0.3 mg/kg) in mice was performed as previously reported [31, 32]. Mice were treated from the 16th to 23rd weeks, once a day for 6 days a week, and at least 30 min before any behavioural testing. All behavioural experimenters were kept blind to genotype and treatment.

## Behavioural tests

### Clasping test

The muscular spasticity of mice was evaluated by the clasping test. The test consisted of suspending the mouse by the tail for 10 s and to score the hindlimb and toe posture. If no limb clasping and normal escape extension is observed, a score of 0 is assigned. When the mouse has a hindlimb with incomplete spread and loss of mobility, but toes with normal spread, a score of 1 is given. If both hindlimbs exhibit incomplete splay and loss of mobility but toes are normally spread, a score of 2 is considered. When the mouse presents hindlimb contraction with curled toes and immobility, a score of 3 is assigned. Finally, if forelimbs and hindlimbs exhibit clasping and are crossed, with curled toes and immobility, a score of 4 is given. All mice were scored by two independent investigators and, if scores differed by more than one point the mouse was rescored. Scores were averaged.

### Rotarod test

The endurance and motor coordination of mice were evaluated by using a rotarod apparatus (Bioseb, Chaville, France) with an acceleration from 4 to 40 rpm. The latency to fall was recorded. Mice were evaluated every two weeks from the 16th to the 22nd week of life. Over the course of the week, the mice underwent 3 days of rotarod test with 3 trials each. The first day was a training day. The mean of the last 2 trials from days 2 and 3 was used for statistical analysis.

### Beam walking test

The beam walking test measures the fine motor coordination and balance of mice. For that, the time spent by each mouse to cross a beam and the number of foot slips were recorded. Mice were trained every two weeks from the 16th to the 22nd week. Mice were trained with 3 trials a day over 3 days every 2 weeks on a 3-cm-diameter beam. On the last day of training, each mouse was tested 3 times on a 2-cm-diameter beam and the data obtained were averaged for statistical analysis.

### Open-field test

The spontaneous activity of mice was examined in the open-field test. The apparatus consisted of four arenas (50 × 50 cm^2^) equipped with infrared (IR) light-emitting diodes squares (50 × 50 cm^2^). The locomotor activity was recorded through an IR-sensitive camera and analysed using the Open-field software (Viewpoint). The open-field session consisted of placing the mouse in the centre of the square and monitoring the distance covered for 10 min. The open-field test was performed every 2 weeks from the 14th to the 22nd week postnatal.

## Immunohistochemistry

23-week-old mice were euthanized by injection with a mix of Xylazine (7.4 mg/kg, Rompun 2% Elanco, Cuxhaven, Germany) and Ketamine (146 mg/kg, Ketamine 1000, Virbac, France). Lumbar spinal cords were dissected and fixed in 4% paraformaldehyde, 1× PBS overnight at 4 °C. Tissues were cryoprotected in 20% sucrose, 1× PBS overnight at 4 °C, then embedded in OCT (CellPath, Newtown, United Kingdom), cryo-sectioned (20 µm) and placed onto SuperFrost-Plus slides (Epredia, Breda, Netherlands). After permeabilization for 15 min in 1× Tris Buffer Saline (TBS) buffer containing 0.1% Triton X-100 (TBST), sections were saturated for 1 h in the saturation buffer (1× TBS, 0.1% Triton X-100, 2% bovine serum albumin), then incubated overnight at 4 °C in the saturation buffer with the following antibodies: anti-neuronal nuclei (NeuN), anti-glial fibrillary acidic protein (GFAP), anti-ionized calcium-binding adapter molecule 1 (IBA1) or anti-vesicular glutamate transporter 1 (VGLUT1) [33] (Table S2). After 3 rinses with 1× TBS, sections were incubated for 1.5 h at room temperature with the saturation buffer containing secondary antibodies: Cy3 anti-mouse, Alexa Fluor 488 Goat IgG anti-rabbit, Alexa Fluor 488 goat IgG anti-chicken or Cy3 anti-rabbit (Table S2). Finally, sections were briefly washed with 1× TBS, incubated for 4 min with DAPI (dilution 1:30,000; ab228549; Abcam, Cambridge, UK) and washed in water. Slides were mounted using FluoroSaveTM Reagent (345789; Merck, Saint-Quentin-Fallavier, France).

## Image acquisition

NeuN, GFAP and IBA1 immunolabelling images were captured using a Zeiss LSM880 Fast Airyscan confocal microscope (Rueil Malmaison, France) equipped with a 20×/0.8 Plan Apo-Chromat (Zeiss, Champigny sur Marne, France) piloted using the Zeiss Black Software. VGLUT1 immunolabelling was acquired using a Leica Thunder (Wetzlar, Germany) equipped with a 20×/0.55 HC PL Flutar (Leica, Wetzlar, Germany) piloted using the LAS X software. Z-stacks (10-µm thick) were analysed using the FIJI software. Motoneurons were identified as large NeuN-immunopositive neurons (soma diameter > 20 µm) in the ventral horn of the spinal cord. They were quantified using Cell Counter FIJI plugins on 3 sections separated by 200 µm per individuals, and means were used for statistical analysis. The glial reactivity in the ventral horn was detected by immunostaining of astrocytes with GFAP antibody or microglia with IBA1 antibody. Quantification of the area occupied by the labelling and the number of cells per mm^2^ throughout the ventral horn was performed with the ImageJ software. For the expression of VGLUT1, the density of VGLUT1-positive puncta in the ventral or the dorsal horn was analyzed with the ImageJ software. For each mouse, three images were acquired and averaged prior to statistical analysis.

## Statistical analysis

Data are expressed as mean ± standard error of the mean (SEM). Statistical analysis was conducted using Prism GraphPad 8.3 software. Before any analysis, a normality test was performed to determine if data followed or not a normal distribution. When a normal distribution was observed, Student’s *t*-test or one-way ANOVA followed by a Tukey’s test was used to compare two groups or more, respectively. In the case of non-parametric distribution, the Mann-Whitney test was used to compare two groups, or the Kruskal-Wallis followed by a Dunn’s test was used for multiple group comparison. For longitudinal analysis, two-way ANOVA with time and treatment as independent factors followed by Dunnett’s test was performed. Statistical significance was set at *P* < 0.05. The statistical data are summarized in Additional file 2.

## Results

### Positive modulation of S1R restores locomotor response of ALS zebrafish

**Fig. 1:**
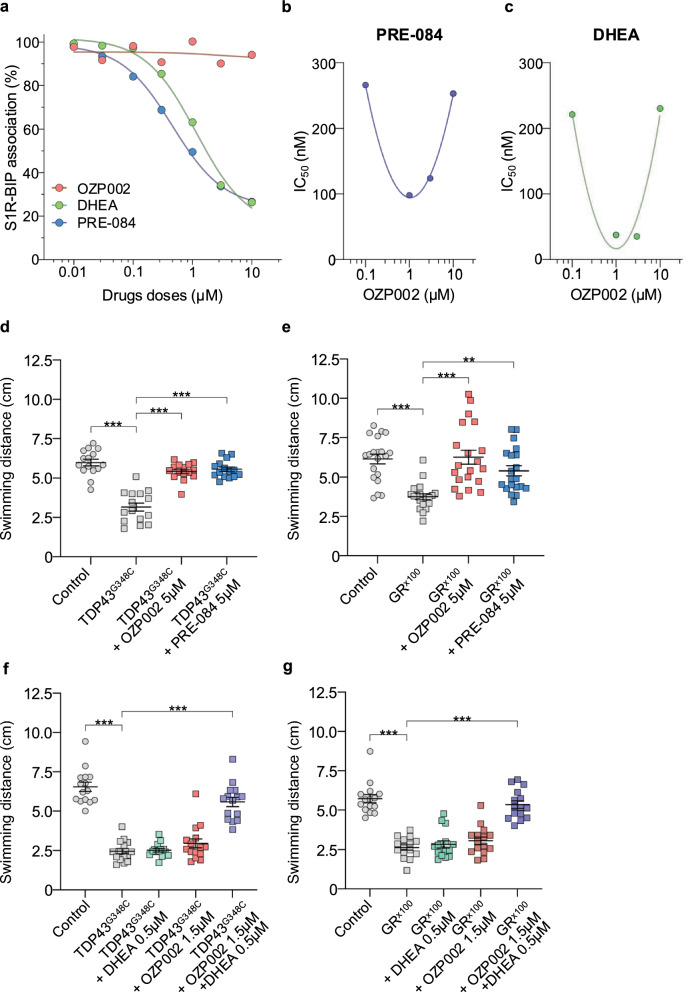
OZP002 and PRE-084 mitigate ALS pathology in zebrafish. **a** S1R/BiP dissociation assay for the reference S1R synthetic agonist PRE-084 (IC_50_ = 461 nM, blue), the endogenous agonist DHEA (IC_50_ = 1169 nM, green), and OZP002 (red). S1R/BiP association was normalised to untreated control. **b**, **c** Impact of OZP002 (0.1–10 µM) on S1R/BiP association after a co-treatment with the agonist PRE-084 or DHEA. Respective IC_50_ values are shown for each OZP002 concentration in a log10 scale. **d** Touch-escape response of control larvae or larvae expressing TDP43^G348C^ alone or treated with OZP002 (5 μM) or PRE-084 (5 μM). Swimming distance in 5 s was measured after mechanical stimulation of the tail fin at 5 dpf. **e** Touch-escape response of control larvae or larvae expressing GR^x100^ alone or treated with OZP002 (5 μM) or PRE-084 (5 μM). **f** Touch-escape response of control larvae or larvae expressing TDP43^G348C^ alone or treated with DHEA (0.5 μM), OZP002 (1.5 μM) or together with DHEA and OZP002 (0.5 + 1.5 μM, respectively). **g** Touch-escape response of control larvae or larvae expressing GR^x100^ alone or treated with DHEA, OZP002 or DHEA + OZP002. Statistical analysis details are shown in Additional file 2, Statistical analyses file (**P < 0.01; *** *P* < 0.001)

OZP002 is a phoshinolactone-based analogue of the antidepressant hydroxybupropion [20]. It was previously reported to positively modulate S1R activity but not by binding the S1R active site [32]. A cellular S1R activity test has been designed by ligand-induced S1R-BiP dissociation. In basal conditions, S1R is associated with the BiP chaperone protein. When S1R is activated by selective agonists, it would be released from BiP. Here we found that OZP002 failed to dissociate BiP from S1R, contrary to the reference synthetic agonist, PRE-084 or the steroid, DHEA, putatively acting as an endogenous S1R ligand (Fig. 1a). The IC_50_ values of DHEA and PRE-084 on S1R-BiP dissociation were 1169 nM and 461 nM, respectively, indicating that the PRE-084 is twice more efficient than the steroid. Addition of OZP002 significantly reduced the IC_50_ values of both PRE-084 (down to 98 nM) and DHEA (down to 35 nM) in a bell-shaped manner (Fig. 1b and c). Noteworthy, the potentiation of S1R-BiP dissociation by OZP002 was more pronounced for DHEA (~ 6.5 fold) than PRE-084 (~ 4.7 fold).

Then, the impact of S1R activators on the locomotor performances of two distinct ALS models of zebrafish was evaluated. Due to the mislocalisation of TDP43 in ALS, both loss of nuclear function and gain of cytoplasmic function are thought to cause the disease [34]. To model the gain of function, human mutant TDP43 (TDP43^G348C^) was expressed in zebrafish larvae. The touch-escape assay was used to quantify the locomotor performances consecutively to a mechanical stimulation at 5 dpf. Larvae expressing mutant but not wild-type TDP43 swam a shorter distance in 5 s compared to the control larvae (Fig. S1a). In contrast, treatment with OZP002 at 5 µM significantly restored the performances of TDP43^G348C^ larvae (Fig. 1d). In comparison, treatment with PRE-084 at the same concentration conferred a similar rescue effect (Fig. 1d).

The efficacy of S1R activators was also evaluated in another ALS zebrafish model that recapitulates a part of the complex pathology mediated by *c9orf72* mutations. One of them is the unconventional non-ATG translation of toxic dipeptides [35]. We chose to express one of the most toxic dipeptide repeats, i.e., glycine-arginine (GR), in zebrafish larvae [24]. A significant decline of swimming distance was observed when 100 GR repeats (GR^x100^) were expressed compared to larvae containing the control construct, i.e., GR^x100^ downstream a stop codon (Fig. S1b). Here again, treatment with OZP002 or PRE-084 at 5 µM was effective in rescuing the locomotor escape response of larvae expressing the *c9orf72* mutation (Fig. 1e). As control, treatment with OZP002 or PRE-084 at 5 µM did not affect the locomotor performances of non-diseased zebrafish (Fig. S1c). To demonstrate that OZP002 is a modulator able to potentiate the impact of endogenous S1R agonists, TDP43^G348C^ or GR^x100^-expressing larvae were co-treated with DHEA and OZP002. DHEA improved locomotor performances of TDP43^G348C^ and GR^x100^ zebrafish at 2 µM but not 5 µM, suggesting a bell-shape dose–response efficacy (Fig. S2). Interestingly, when two infraliminal doses of DHEA (0.5 µM) and OZP002 (1.5 µM) were combined, a significant improvement in swimming distance of TDP43^G348C^- and GR^x100^-expressing larvae was observed (Fig. 1f, g).

Finally, to compare the potential toxicity of OZP002 and PRE-084, 4-dpf zebrafish larvae were exposed for 24 h to increasing doses of both S1R activators. PRE-084 started to show signs of toxicity at 22 µM with the appearance of a few malformed larvae (Fig. S3a, b) and a decrease in the spontaneous activity of 5-dpf larvae (Fig. S3c). In contrast, after an exposure to OZP002, no toxicity was detected at concentrations up to 105 µM.

## S1R conferred protection through NRF2 signalling cascade

**Fig. 2:**
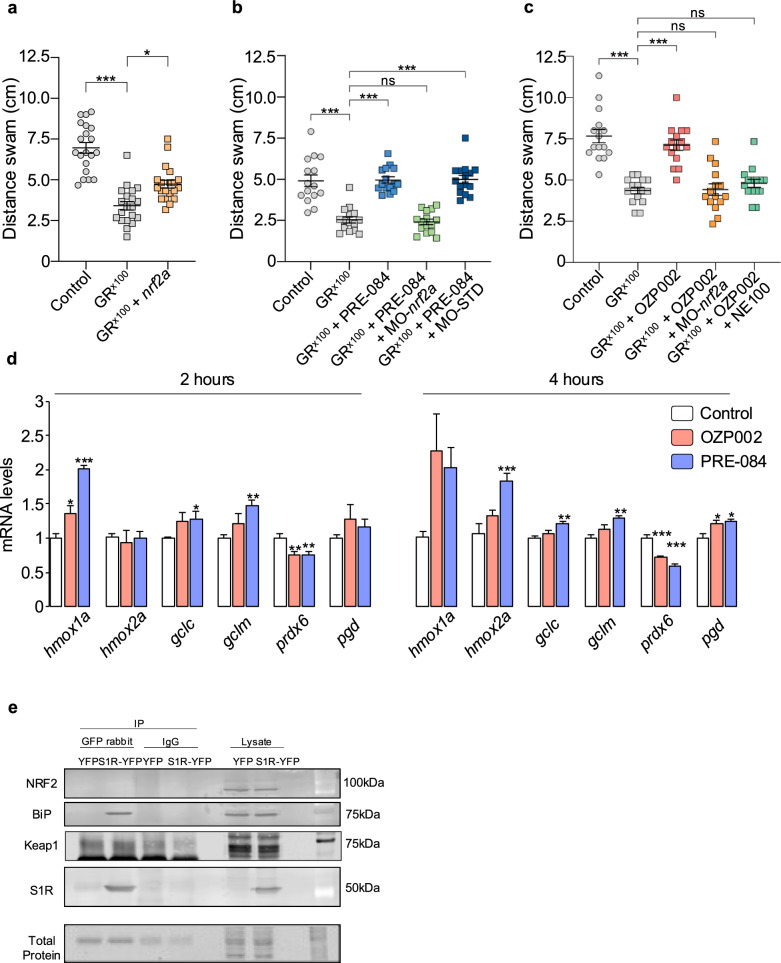
The beneficial effect of S1R activation is partly mediated by the NRF2 cascade.** a** Touch-escape responses of control larvae, larvae expressing GR^x100^ alone or GR^x100^ with *nrf2a*. Data from 20 larvae were averaged and presented as mean ± SEM.** b** Touch-escape responses of control larvae, larvae expressing GR^x100^ alone with or without PRE-084 (5 μM) treatment, or larvae expressing GR^x100^ with morpholinos (*nrf2a* or standard control morpholinos) and treated with PRE-084. Data from 15 larvae were averaged and presented as mean ± SEM.** c** Touch-escape responses of control larvae, larvae expressing GR^x100^ alone, GR^x100^ larvae treated with OZP002 or OZP002 + NE100, or larvae expressing GR^x100^ and MO-*nrf2a* and treated with OZP002. Data from 15 larvae were averaged and presented as mean ± SEM.** d** mRNA expression of NRF2 targets: *gclc*, *gclm*, *hmox1, hmox2, prdx6* and *pgd*, quantified in 5-dpf larvae treated with OZP002 (5 μM) or PRE-084 (5 μM) for 2 or 4 h. Data are presented as mean ± SEM. Analysis was performed on 5 tubes containing 8 larvae. **e** Co-immunoprecipitation of S1R-YFP and NRF2 in S1R-KO N2a cells overexpressing YFP or S1R-YFP. Immunoprecipitation of YFP with antibodies or IgG produced in rabbit and analysis of the co-immunoprecipitants through western blot analysis. No S1R interaction was observed with NRF2 or Keap1. Self-IP and BiP immunoprecipitation are noted. Statistical analysis details are shown in Additional file 2, Statistical analyses file (ns: not significant; *P < 0.05; **P < 0.01; ***P < 0.001)

Our previous data suggested that the protective effects of PRE-084 may involve the NRF2 signalling cascade [18]. NRF2 is a key transcription factor that regulates protection against oxidative stress and inflammation. Of interest, we previously reported that increasing NRF2 levels mitigated locomotor defects in TDP43^G348C^-expressing larvae. To provide further evidence, we first determined if it was also the case for C9orf72 larvae expressing GR^x100^. Zebrafish have duplicate *nrf2* genes (*nrf2a* and *nrf2b*). However, Nrf2a was reported to be the most similar to human NRF2 in terms of genetic sequence and function [26]. Microarray studies revealed that Nrf2a and Nrf2b regulate distinct gene sets and the impact of *nrf2a* knockdown is more consistent with the well-known role of vertebrate NRF2 proteins as a transcriptional activator [26]. Here we demonstrated that overexpressing *nrf2a* ameliorated the impaired escape locomotor response of GR^x100^ larvae (Fig. 2a). Downregulating *nrf2a* expression with a morpholino [26] had no impact on the locomotion of GR^x100^ larvae (Fig. S4a). Importantly, in the presence of the *nrf2a* morpholino, OZP002 and PRE-084 failed to alleviate the locomotor defects of GR^x100^ larvae (Fig. 2b, c). To assess potential off-target effects of morpholinos, a standard control morpholino was also tested with PRE-084. In this case, treatment with PRE-084 remained effective to rescue the locomotor defects due to GR^x100^ (Fig. 2b). As previously observed for PRE-084 [18], the S1R antagonist NE-100 also prevented the beneficial effect of OZP002, thus confirming the S1R selectivity of OZP002 (Fig. 2c). This demonstrated that the beneficial effects of both S1R activators involve the NRF2 signalling cascade.

NRF2 is known to stimulate antioxidant defence. Thus, we compared the impacts of OZP002 and PRE-084 on several antioxidant genes. Analysis was performed on 5-dpf larvae after treatment for 2 h or 4 h. PRE-084 stimulated gene expression of heme oxygenase (*hmox1a* and *hmox2a*), NADPH-producing enzyme 6-phosphogluconate dehydrogenase (*pgd*) as well as the catalytic (*gclc*) and the modifier subunits (*gclm*) of glutamate-cysteine ligase (Fig. 2d). In a lesser extent, OZP002 tended toward a similar impact on those genes but with a significant increase only for *hmox1a* mRNA. In contrast, both OZP002 and PRE-084 downregulated the mRNA expression of peroxiredoxin 6 (*prdx6*) (Fig. 2d). The downregulation of *prdx6* gene is in accordance with the direct regulation by S1R previously established [36]. To further investigate a potential direct interaction between S1R and NRF2 or its negative modulator Keap1, we performed an immunoprecipitation assay. For that purpose, we used S1R-KO N2a cells with a stabilised transfection with YFP or S1R-YFP. The advantage of using S1R-YFP in S1R-KO is to have a homogenous population of higher expression level of S1R-YFP without influence of endogenous S1R. Although as expected we observed the co-immunoprecipitation of BiP with S1R, we did not observe NRF2 or Keap1 bands after precipitating S1R (Fig. 2e, Fig. S4b). Our data indicated that S1R does not directly interact with NRF2 or Keap1 in our experimental condition.

## S1R activation mitigates locomotor defects of ALS mice expressing TDP43^A315T^

**Fig. 3:**
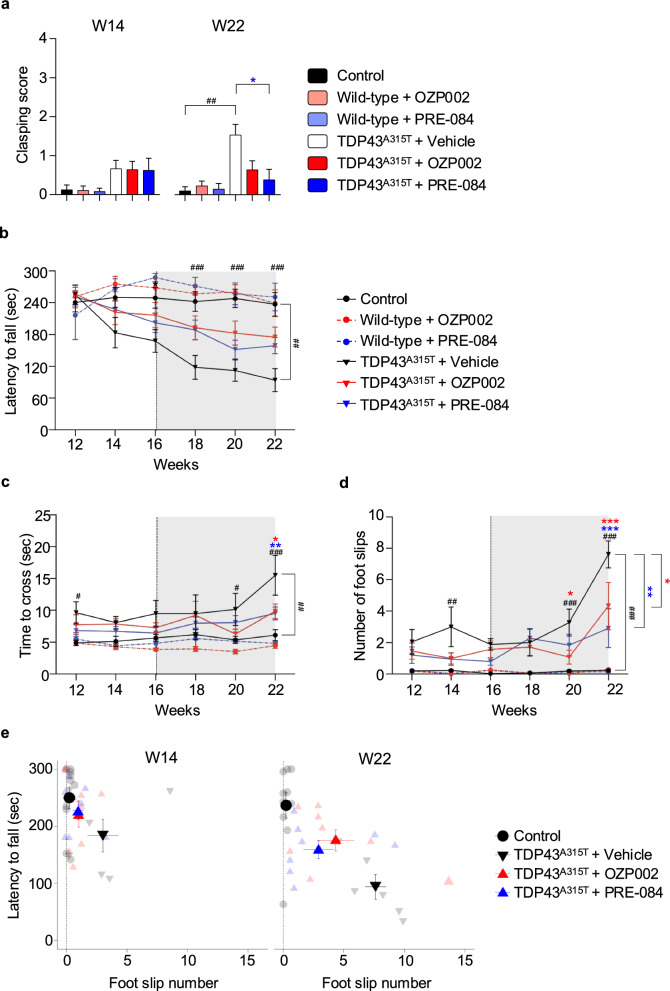
S1R activation ameliorates the locomotor deficits of TDP43^A315T^ transgenic mice. **a** Clasping performance (scored from 0 to 4) measured on wild-type mice and TDP43^A315T^ transgenic mice treated with vehicle saline solution, OZP002 (0.7 mg/kg), or PRE-084 (0.3 mg/kg). Treatments are administered from age of 16 weeks. **b** Rotarod performance (latency to fall) between 12 and 22 weeks of age of either wild-type mice or TDP43^A315T^ transgenic mice treated with saline solution, OZP002 (0.7 mg/kg), or PRE-084 (0.3 mg/kg). **c, d** Beam walking performance (time to cross and number of foot slips) on 2-cm-diameter beam was analysed between 12 and 22 weeks of age on wild-type or TDP43^A315T^ mice treated with saline solution, OZP002 or PRE-084. **e** Locomotor performances obtained at 14 and 22 weeks of age with the rotarod test (latency to fall) plotted versus the beam walking test (number of foot slips) on wild-type or TDP43^A315T^ mice treated with saline solution, OZP002 or PRE-084. Data from 6 to 10 mice were averaged and presented as mean ± SEM. For the clasping performance, data were assessed by Kruskal–Wallis test followed by Dunn’s test. For the Rotarod and Beam walking performances, statistical analysis details are shown in Additional file 2, Statistical analyses file (*P < 0.05; ** *P* < 0.01; *** *P* < 0.001 vs TDP43^A315T^ + saline solution; ^#^ *P* < 0.05; ^##^ *P* < 0.01; ^###^ *P* < 0.001 vs Control)

So far, the efficacy of S1R activation on ALS rodent models has been evaluated by using agonists and solely on the *SOD1* mutation context. For the first time, we here set up analyses on the locomotor deficits of TDP43^A315T^ mice expressing mutant TDP43 under the control of the murine prion promoter [37]. Because those transgenic mice were previously reported to prematurely die due to intestinal obstruction [29], they were fed with jellified food [30]. Accordingly, TDP43^A315T^ female mice survived up to the 23rd week without dramatic body weight loss (Fig. S5a). S1R activator OZP002 or PRE-084, or vehicle solution, was administered by intraperitoneal injection in wild-type and TDP43^A315T^ littermate female mice from the age of 16 weeks. No overt secondary effects were observed in the treated mice as aggressiveness, abnormal behaviour or coat condition. To determine whether treatments affected motor functions, four motor behavioural assessments were conducted: limb-clasping score, spontaneous locomotor activity in the open field test, rotarod latency to fall, crossing time and number of missteps on a beam. Compared to control mice, TDP43^A315T^ mice progressively displayed a clasping phenotype with a score that reached 1.5 versus 0.1 at 22 weeks of age (Fig. 3a). Treatment with S1R activators reduced the clasping score with a significant outcome for PRE-084 (Fig. 3a). No change of the spontaneous locomotor activity was observed between control mice and mutant mice (Fig. S5b). TDP43^A315T^ mice showed a progressive decline of their ability to remain on the accelerating rotarod compared to controls (Fig. 3b). Treatment with OZP002 or PRE-084 tended to alleviate this defect but none reached statistical significance. Interestingly, fine motor coordination analysis using the beam walking test revealed that TDP43^A315T^ mice had impaired balance, but that treatment with PRE-084 or OZP002 significantly improved their performances (Fig. 3c, d). Both the time to cross the beam and the number of foot slips were reduced at 22 weeks of age. To better visualise the impact of S1R activators on TDP43 mice, the motor performances on the rotarod and beam walking test were summarized on the same graph (Fig. 3e). While locomotor performances of TDP43^A315T^ mice sharply worsened between 14 and 22 weeks, the deficit was less pronounced when S1R was activated. As a control, treatment of wild-type mice with PRE-084 or OZP002 did not affect the clasping score, the spontaneous activity on the open field, or the locomotor performances on the rotarod or in the beam walking test (Fig. 3, Fig. S5b).

## S1R activation prevents motor neuron loss in the spinal cord of TDP43^A315T^ mice

**Fig. 4:**
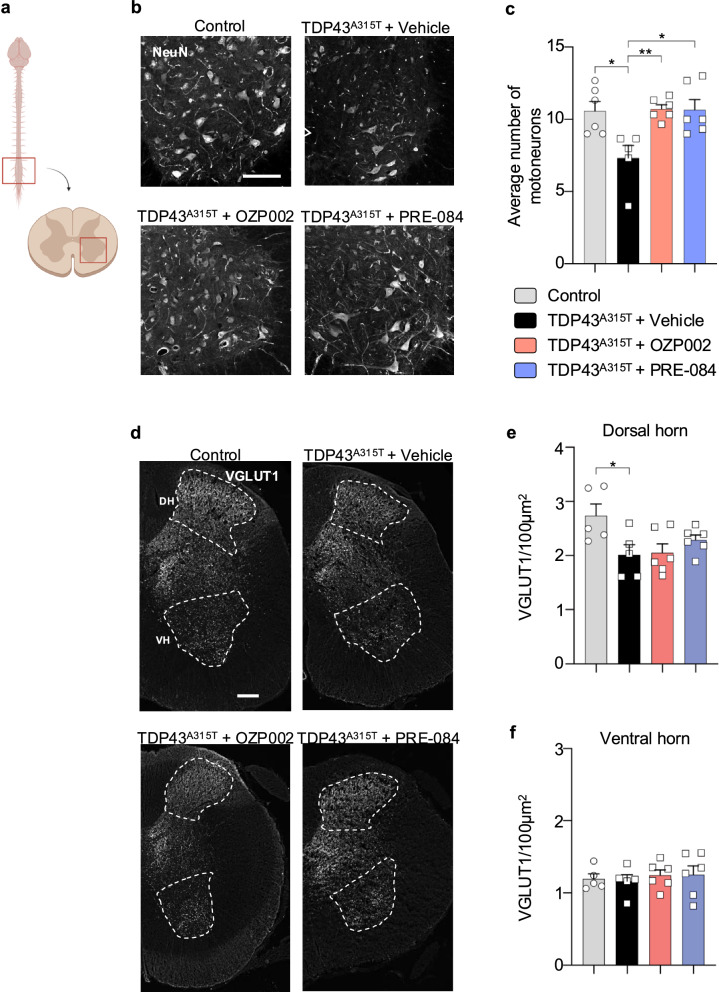
OZP002 and PRE-084 prevent motoneuron loss. **a** Spinal cords were collected from euthanised mice and the lumbar region innervating hindlimbs was conserved for immunohistochemistry. **b** Representative NeuN immunostainings in the lumbar spinal cords of wild-type mice treated with saline solution (Control), and TDP43^A315T^ transgenic mice treated with saline solution, OZP002 (0.7 mg/kg), or PRE-084 (0.3 mg/kg). Scale bar: 90 μm. **c** Quantification of the numbers of NeuN^+^ motoneurons in the lumbar spinal cord of mice. NeuN immunoreactive motoneurons were counted based on soma size criteria (diameter > 20 µm) and morphology. Data are the mean of the number of motoneurons averaged from 3 consecutive lumbar slices per mouse. **d** Representative staining of VGLUT1 in the lumbar spinal cords of Control mice, and TDP43^A315T^ mice treated with saline solution, OZP002 (0.7 mg/kg), or PRE-084 (0.3 mg/kg). DH: dorsal horn, VH: ventral horn. Scale bar: 110 μm.** e** Quantification of the density of VGLUT1-positive puncta per 100 μm^2^ in the dorsal horn of the lumbar spinal cord. **f** Quantification of the density of VGLUT1-positive puncta per 100 μm^2^ in the ventral horn of the lumbar spinal cord. Analysis was performed on 3 consecutive lumbar slices per mouse, *n* = 5–6 mice. Statistical analysis details are shown in Additional file 2, Statistical analyses file (*P < 0.05; **P < 0.01)

Histological analysis of the lumbar spinal cord sections revealed a significant loss of NeuN-positive motoneurons in TDP43^A315T^ mice (Fig. 4b, c). The mean number of motoneurons in the spinal cord section was 11 in control mice but was reduced to 7 in TDP43^A315T^ mice at 23 weeks of age (Fig. 4c). The neurodegeneration was prominently prevented by the intraperitoneal administration of OZP002 or PRE-084 (Fig. 4b, c). Spinal synaptic terminals strongly express the vesicular glutamate transporter, VGLUT1. They mainly originate from the primary sensory afferents, as well as descending cortical neurons and spinal interneurons. In a previous study, a loss of VGLUT1-positive synapses was reported in TDP43^A315T^ mice [38]. Here, we found a significant decrease in the density of VGLUT1-immunopositive inputs in the dorsal part of the spinal grey matter but not in the ventral part of TDP43^A315T^ versus control mice (Fig. 4d–f). The treatments with S1R activators failed to diminish this loss of VGLUT1 in the dorsal part.

## S1R activation reduces glial reactivity in TDP43^A315T^ mice

**Fig. 5:**
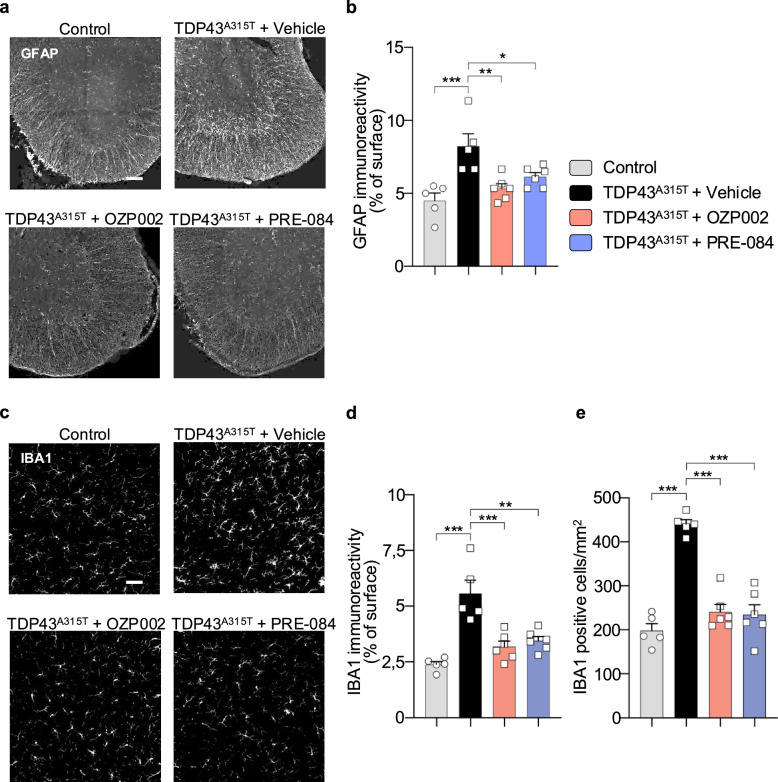
OZP002 and PRE-084 reduce glial reactivity. **a** Representative GFAP immunostainings in the lumbar spinal cords of either wild-type mice treated with saline solution (Control), or TDP43^A315T^ transgenic mice treated with saline solution, OZP002 (0.7 mg/kg), or PRE-084 (0.3 mg/kg). Scale bar: 110 μm. **b** Astrocyte reactivity was measured as the percent of surface occupied by GFAP immunolabelling. **c** Representative IBA1 immunostainings in the lumbar spinal cords of Control mice, or TDP43^A315T^ transgenic mice treated with saline solution, OZP002 (0.7 mg/kg), or PRE-084 (0.3 mg/kg). Scale bar: 110 μm.** d** Microglial reactivity was measured as the percent of surface occupied by IBA1 immunolabelling.** e** Quantification of numbers of IBA1^+^ microglial cells in the lumbar spinal cords of mice. Analysis was performed on 3 consecutive lumbar slices per mouse, *n* = 5–6 mice. Statistical analysis details are shown in Additional file 2, Statistical analyses file (*P < 0.05; ** *P* < 0.01; *** *P* < 0.001)

TDP43^A315T^ mice display a strong glial reaction in the spinal cord [39]. Astroglia and microglia were detected by GFAP and IBA1 immunostainings, respectively, in the ventral horn (Fig. 5a, c). Spinal sections of TDP43^A315T^ mice showed an 82% increase in surface occupied by GFAP-immunostained astrocytes (Fig. 5b). In contrast, treatment with OZP002 or PRE-084 effectively reduced astroglial reaction; in those conditions GFAP-immunostained surfaces remained similar to those observed for controls (Fig. 5b). Microglia immunostaining was also augmented in TDP43^A315T^ mice (Fig. 5c), resulting in an increase in both IBA1-immunostained area and microglial cell density (Fig. 5d, e). Of interest, treatment with OZP002 or PRE-084 restored both the surface and the density of IBA1 labelling, indicating that S1R activation prevented microglial reaction in the spinal cord.

## Discussion

**Fig. 6:**
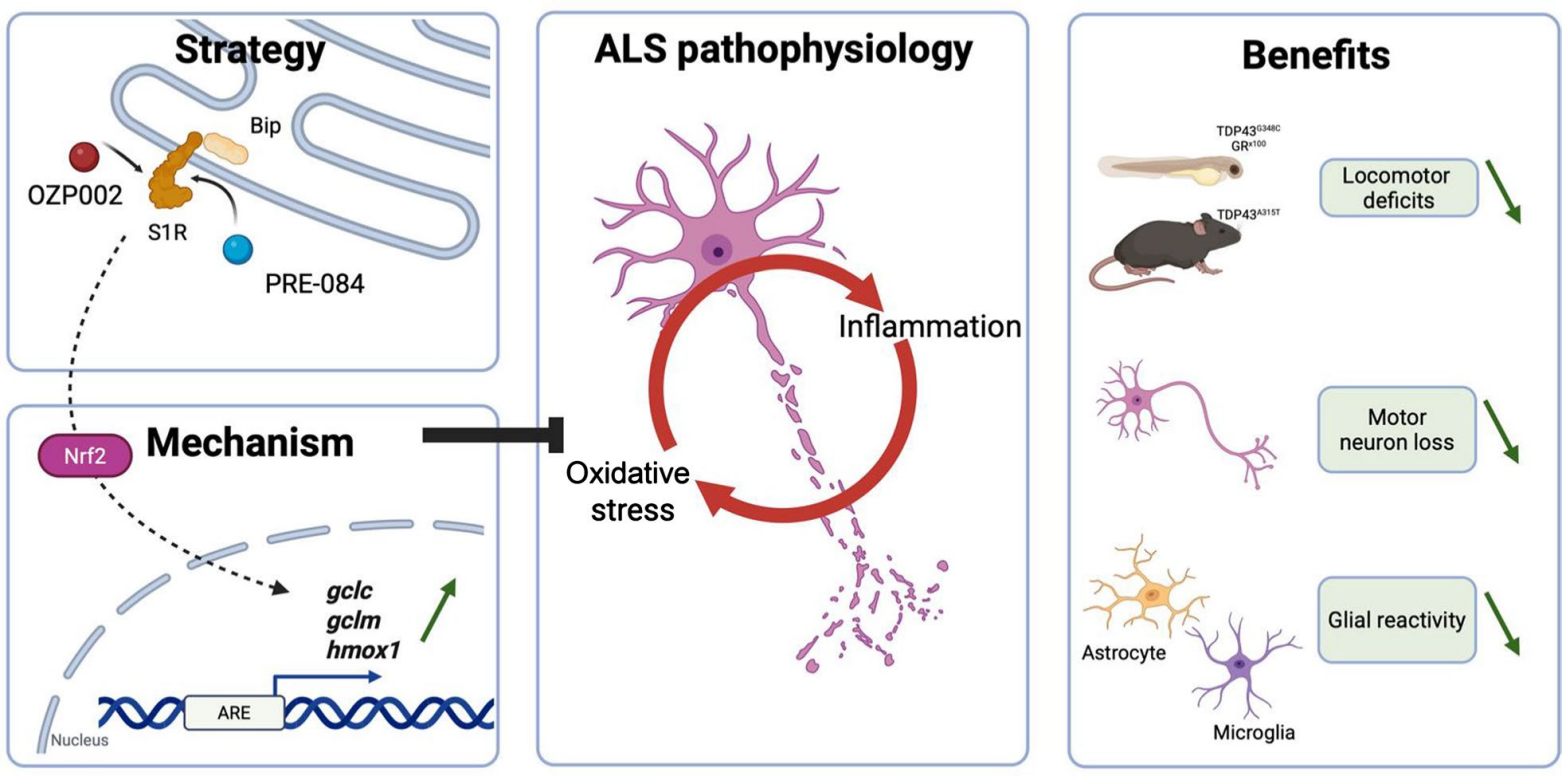
A schematic representation of the therapeutic approach by targeting S1R with OZP002 or PRE-084. This increases the NRF2-mediated response, reducing oxidative stress and inflammation. This strategy alleviates motor deficits, preserves motor neurons, and limits glial reactivity. Figure created with BioRender.com

The therapeutic value of S1R activation in ALS has been under the spotlight for more than a decade. However, while there is no doubt that S1R activation by agonists confers neuroprotection against the expression of mutated SOD1 [13–16], little evidence has been provided in other ALS contexts. Nevertheless, the S1R agonist, Pridopidine, has recently been introduced into phase 3 clinical trials following encouraging results in patients with ALS [40]. Here, we demonstrate the high efficacy of S1R activation in two vertebrate models that recapitulate the toxic gain-of-function triggered by two key proteins implicated in ALS: TDP43 and C9orf72. Mislocalisation of TDP43 is a pathological hallmark in up to 97% of ALS patients and 45% of FTLD cases [5, 41]. The hexanucleotide repeat expansions in *C9orf72* gene is the most common cause known to result in ALS or FTLD [3, 4]. We here provide strong evidence that S1R activation alleviates not only the locomotor deficits of zebrafish recapitulating toxic gain-of-function of TDP43 or C9orf72, but also locomotor impairment, neuronal loss and glial reaction in TDP43^A315T^ transgenic mice (Fig. 6).

To date, only synthetic S1R agonists have been studied for their efficacy in ALS condition. PRE-084, SA4503 and Pridopidine have all been reported to confer protection in SOD1^G93A^ mice [15–17]. We have here chosen to evaluate a different class of compounds in drug discovery, namely modulators. Unlike the reference agonist PRE-084, they induce conformational changes in S1R by binding to a site other than the orthosteric site where natural endogenous agonists bind. In the case of S1R that is not a classical receptor, they offer an alternative approach to modulate its chaperone activity. Of interest, positive modulators potentially achieve the same efficacy as agonists, as confirmed for S1R modulators in vivo [19, 32], but they generally cause fewer adverse effects due to the structural diversity of modulation sites. OZP002 does not inhibit binding of the classic prototypical orthosteric ligand, ( +)-pentazocine, but rather leads to a slight non-significant increase of its binding [32]. Accordingly, OZP002 failed to dissociate BiP from S1R. However, OZP002 can increase the S1R-BiP dissociation induced by the S1R agonist Igmesine [32] and in our study it can reduce the IC_50_ values of the reference agonist PRE-084 and the steroid DHEA. Thus, our data confirm that OZP002 behaves as a positive modulator, as initially characterised [32].

While it is well known that modulators of S1R affect processes involved in the pathophysiology of memory, cognitive disorders, depression and seizures, their impact on locomotor defects has been relatively underexplored [19]. Our analyses reveal that OZP002 mitigates locomotor deficits in ALS models, demonstrating an efficacy comparable to that of the PRE-084. A question is whether or not OZP002 requires the presence of endogenous agonists to be efficient on locomotor behaviour [42]. Our data do not allow us to respond to this possibility. However, we demonstrate that OZP002 enhances the DHEA-induced improvement of locomotor function of ALS zebrafish.

The neuroprotection conferred by S1R activation may involve several mechanisms that are challenged in the ALS context. Among them, S1R may mitigate oxidative/ER stress and inflammation that form a vicious cycle in ALS. We have previously shown that PRE-084 treatment activates NRF2 target genes and that increasing NRF2 levels significantly rescued locomotor escape response of TDP43-expressing zebrafish [18]. Treatment with OZP002 also appeared to stimulate the NRF2 cascade but to a lesser extent, suggesting that S1R activation confers protection at least in part through the NRF2 pathway (Fig. 6). Among NRF2 target genes, *hmox1* was the most significantly upregulated by OZP002 and PRE-084. HMOX-1 converts Heme into three bioactive products, namely, free iron, carbon monoxide and biliverdin, which play crucial roles in preventing inflammation and oxidative stress [43]. Biliverdin/bilirubin reduces pro-inflammatory responses through the NF-κB pathway [44] and inhibits toll-like receptor 4 pro-inflammatory signalling [45]*.* Carbon monoxide at low physiological doses is implicated in anti-inflammatory effects notably through overexpression of interleukin IL-10 [46, 47]. The beneficial impact of NRF2 in the context of ALS is underscored by our data showing that increasing Nrf2a levels enhanced the locomotor escape response of zebrafish expressing the GR^x100^ and by our previous observation with mutated TDP43 [18]. Moreover, PRE-084 and OZP002 treatments were found ineffective on the locomotor defects in the presence of *nrf2a* morpholino, further highlighting the role of NRF2 in S1R-mediated neuroprotection.

The precise mechanism by which S1R stimulates the NRF2 cascade is still elusive. In physiological condition, NRF2 binds to Keap1, a substrate adaptor protein for the E3 ubiquitin ligase Cullin3. This interaction results in the ubiquitination of NRF2 and its proteasomal degradation [48]. In oxidative stress condition, Keap1 configuration is altered by adduction or oxidation of its cysteine residues [49], leading to the dissociation of NRF2 and its translocation into the nucleus. Previous studies have investigated a possible regulation of the NRF2 protein turnover by S1R. While NRF2 was proposed to directly interact with S1R in cone photoreceptor 661w cell line [50] and in kidney-derived HK-2 cells [51], no coimmunoprecipitation of NRF2 was detected in CHO cell line transiently transfected with Flag-S1R [52]. To further investigate the potential interaction between S1R and NRF2 or its negative regulator Keap1, we chose to use N2A cells knock-out for S1R and transfected with S1R tagged with YFP. We detected no direct interaction between S1R and NRF2 or Keap1 in this strict experimental condition. Intriguingly, Cullin3 was also found immunoprecipitated with S1R in 661w photoreceptor cells, further complicating the understanding of the interaction between S1R and the NRF2 axis [53]. Another possibility is that S1R regulates NRF2 nuclear translocation. Recent analyses revealed that S1R activation may increase NRF2 protein levels and nuclear translocation in rat myocardia [54]. However, a completely opposite mechanism was proposed in kidney cells during diabetic nephropathy, where S1R reduced NRF2 phosphorylation and inhibited translocation in the nucleus [51]. More work is still needed to decipher how S1R activates NRF2 signalling. In future, an explanation could benefit from the use of unbiased omics approaches.

The most striking finding of the present study was that OZP002 and PRE-084 prevented locomotor defects, degeneration of spinal motor neurons, and astroglial and microglial activation in TDP43^A315T^ transgenic mice. These data reinforce the idea that S1R may be beneficial more generally in ALS and not just in the context of *SOD1* mutations. While S1R activation prevented the loss of motor neurons in the ventral spinal horn, no significant amelioration was observed for the loss of VGLUT1-positive synapses in the dorsal horn. This may be due to the particular localisation of S1R in the spinal cord, which is more enriched in ventral horn motoneuron cell bodies [55]. Astrocytes and microglia release reactive oxygen species and pro-inflammatory cytokines, contributing to neuronal death. Glial cells also express S1R and its activation may have direct beneficial effects. Activation of S1R has previously been shown in vitro to reduce oxidative stress and inflammation in cultured astrocytes with lipopolysaccharide treatment through the NRF2/HMOX-1 pathway [56]. Similarly, S1R modulates the migration and inflammatory response of cultured microglia, by increasing the intracellular calcium [57].

## Conclusion

Our data highlight the high therapeutic value of S1R activation in previously untested forms of ALS other than in the context of mutant SOD1 overexpression. However, a limitation remains that our models only recapitulate part of the complex pathology, i.e., toxic gain-of-function mutation of TDP43 or C9orf72. It would be interesting to extend in the future the analyses on the loss of nuclear function of both proteins or the cytoplasmic overexpression of wild-type TDP43. Nevertheless, for the first time, we demonstrate in different preclinical models that positive modulators of S1R can achieve the same level of efficacy as a direct orthosteric activator. Thus, our data open a new avenue for drug design of safe and specific molecules to mitigate ALS. We also confirm that the beneficial activity is at least in part mediated by activation of the NRF2 signalling pathway. However, the mechanisms through which the NRF2 cascade is activated via S1R activation remain to be understood.

## Supplementary Information

Additional file 1  **Figure S1**. Validation of ALS models and safety of S1R activators on the touch-escape response. **Figure S2**. DHEA prevents locomotor deficits of TDP43G348C- and GRx100- expressing larvae. **Figure S3**. Acute toxicity assay in zebrafish treated with increasing concentrations of OZP002 or PRE-084. **Figure S4**. Impact of nrf2a morpholino on the touch-escape response and immunoprecipitation analysis using mouse antibodies. **Figure S5**. PRE-084 and OZP002 do not modify weight evolution and spontaneous locomotor activity. **Table S1**. Primers used for PCR and RT-qPCR. **Table S2**. List of antibodies.

Additional file 2 Statistical analyses file.

Additional file 3 Uncropped western blots for Figures 2e and S4b.

## Data Availability

The datasets generated and/or analysed during the current study are available from the corresponding author on reasonable request.

## Abbreviations

ALS: Amyotrophic lateral sclerosis
BiP: Binding immunoglobulin protein
C9orf72: Chromosome 9 open reading frame 72
DHEA: Dehydroepiandrosterone
ER: Endoplasmic reticulum
FTLD: Frontotemporal lobar degeneration
GFAP: Glial fibrillary acidic protein;
GR^x100^: 100 Repeats of glycine-arginine
IBA1: Ionized calcium-binding adapter molecule 1
Keap1: Kelch-like ECH-associated protein 1
NE-100: 4-Methoxy-3-N,N-dipropylbenzeneethanamine
HMOX: Heme oxygenase
NeuN: Neuronal nuclei
NRF2: Nuclear factor erythroid-2 related factor 2
PEG-400: Polyethylene glycol 400
PRDX6: Peroxiredoxin-6
qPCR: Quantitative real-time polymerase reaction chain
S1R: Sigma-1 receptor
SOD1: Superoxide dismutase 1
TDP43: Tar-DNA binding protein 43 kDa
VGLUT1: Vesicular glutamate transporter 1
