# Supplementary material for: Positive modulation of sigma-1 receptor: a new weapon to mitigate disease progression in amyotrophic lateral sclerosis
Source: Transl Neurodegener. 2025 Dec 15;14:68. doi: 10.1186/s40035-025-00527-z (PMC12703938; doi:10.1186/s40035-025-00527-z)
Supplement: Supplementary file 1 — Additional file 1 Figure S1. Validation of ALS models and safety of S1R activators on the touch-escape response. Figure S2. DHEA prevents locomotor deficits of TDP43G348C- and GRx100- expressing larvae. Figure S3. Acute toxicity assay in zebrafish treated with increasing concentrations of OZP002 or PRE-084. Figure S4. Impact of nrf2a morpholino on the touch-escape response and immunoprecipitation analysis using mouse antibodies. Figure S5. PRE-084 and OZP002 do not modify weight evolution and spontaneous locomotor activity. Table S1. Primers used for PCR and RT-qPCR. Table S2. List of antibodies. [file 40035_2025_527_MOESM1_ESM.pdf]

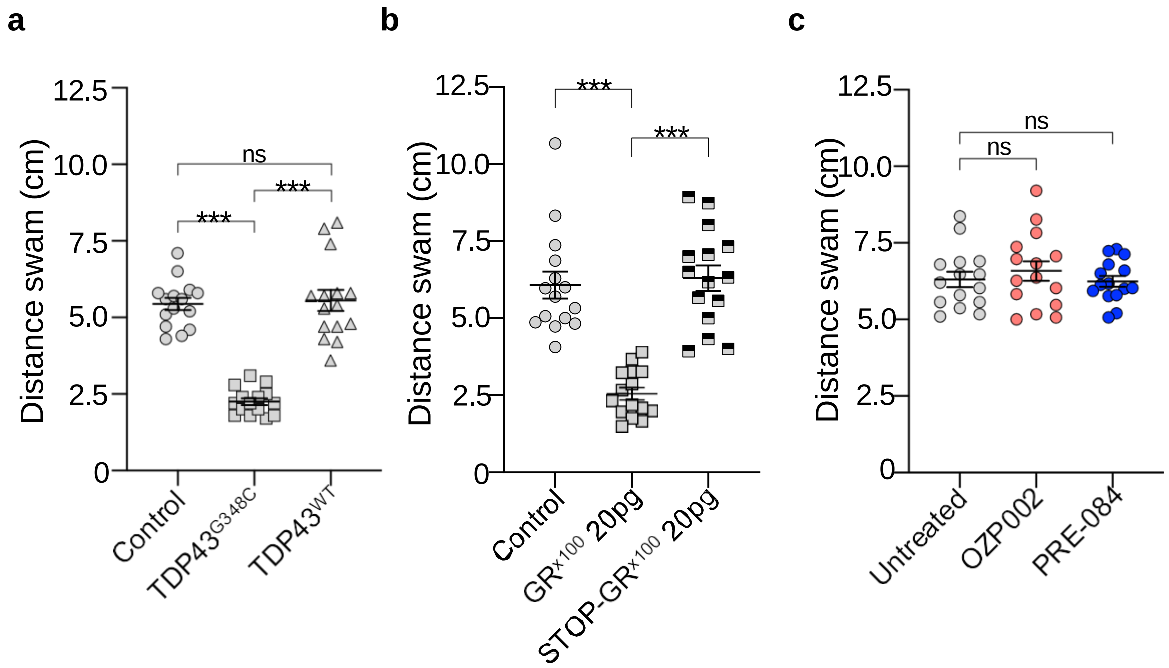

Figure S1. Validation of ALS models and safety of S1R activators on the touch-escape response. **a** Touch-escape response of control larvae, larvae expressing $TDP43^{G348C}$ alone or $TDP43^{WT}$. Data from 15 larvae were averaged and presented as mean ± SEM. Statistical analysis was performed using one-way ANOVA followed by Tukey's test (****P* < 0.001; ns: not significant). **b** Touch-escape response of control larvae, larvae expressing GR^{x100} alone or STOP-GR^{x100}. Statistical analysis was performed using one-way ANOVA followed by Tukey's test (****P* < 0.001). **c**. Touch-escape response of untreated control larvae and larvae treated with OZP002 (5 µM) or PRE-084 (5 µM). Statistical analysis was performed using one-way ANOVA followed by Tukey's test (ns: not significant).

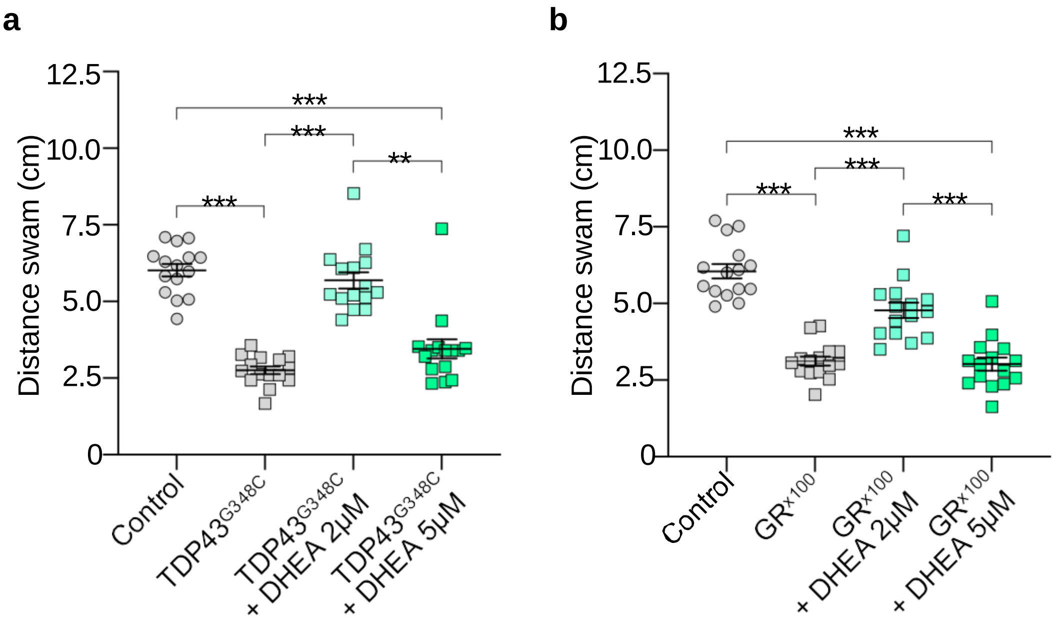

Figure S2. DHEA prevents locomotor deficits of TDP43^{G348C}- and GR^{x100}- expressing larvae. **a** Touch-escape response of control larvae or larvae expressing TDP43^{G348C} alone or treated with DHEA 2 µM or 5 µM. Data from 15 larvae were averaged and presented as mean ± SEM. Statistical analysis was performed using one-way ANOVA followed by Tukey's test (***P < 0.01; *** P < 0.001). **b** Touch-escape response of control larvae or larvae expressing GR^{x100} alone or treated with DHEA 2 µM or 5 µM. Statistical analysis was performed using one-way ANOVA followed by Tukey's test (***P < 0.001).

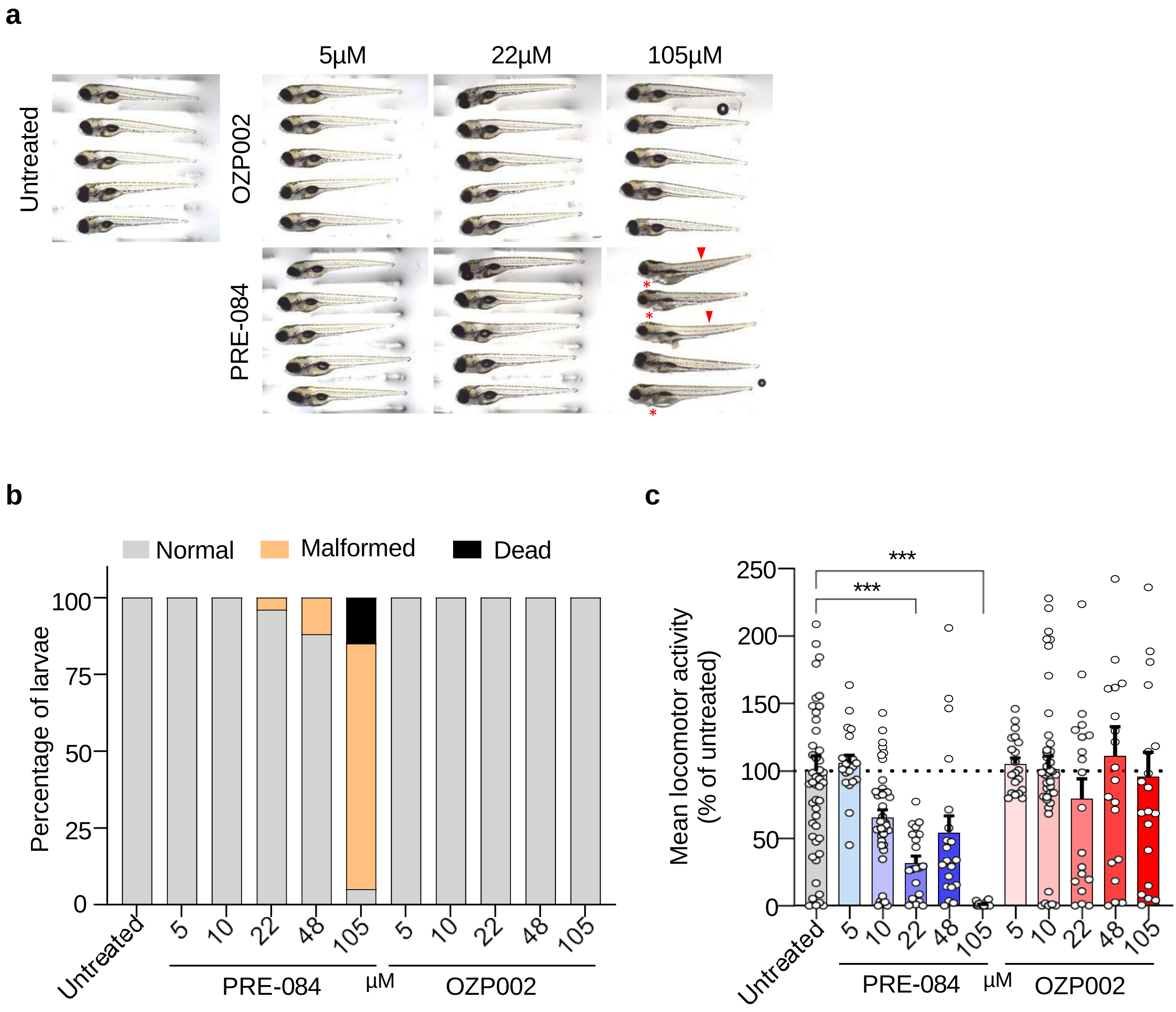

Figure S3. Acute toxicity assay in zebrafish treated with increasing concentrations of OZP002 or PRE-084. a Dose-response representative toxicity of OZP002 and PRE-084 using FET assay on zebrafish larvae following a 24 h exposure. Larvae were reared at 5-22-105 µM od PRE-084 or OZP002. **b** Detailed dose-response observations of FET assay. Larvae were considered malformed if they exhibited cardiac oedema, notochord curvature or prominent jaw, they were considered dead if they presented a consistent lack of heartbeat or body coagulation. **c** Average basal locomotor activity normalized to untreated condition following a 24 h exposure to increasing doses of PRE-084 and OZP002 (untreated n = 48, treated n = 24). Kruskal-Wallis statistical analysis was performed followed by Dunn's multiple comparison test (***P <0.001).

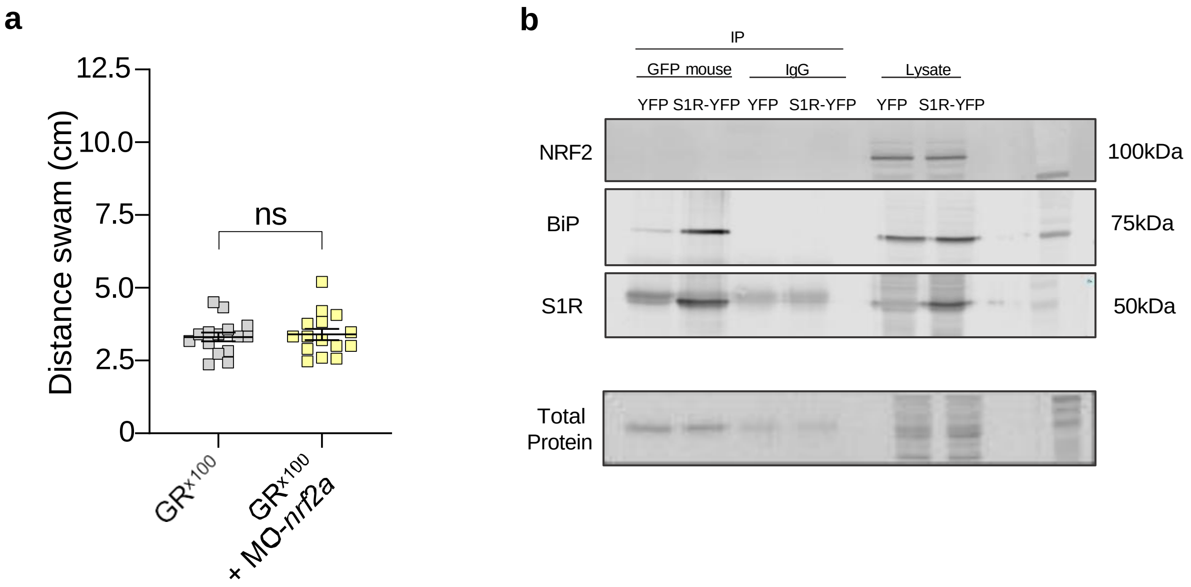

Figure S4. Impact of *nrf2a* morpholino on the touch-escape response and immunoprecipitation analysis using mouse antibodies. a Touch-escape response of larvae expressing GR^{x100} alone and GR^{x100} with MO-*nrf2a*. Data from 15 larvae were averaged and presented as mean ± SEM. Statistical analysis was performed using one-way ANOVA followed by Tukey's test (ns: not significant). **b** Immunoprecipitation of YFP or S1R-YFP with antibodies or IgG produced in mouse and analysis of the co-immunoprecipitants through western blot using antibodies produced in rabbit.

a

b

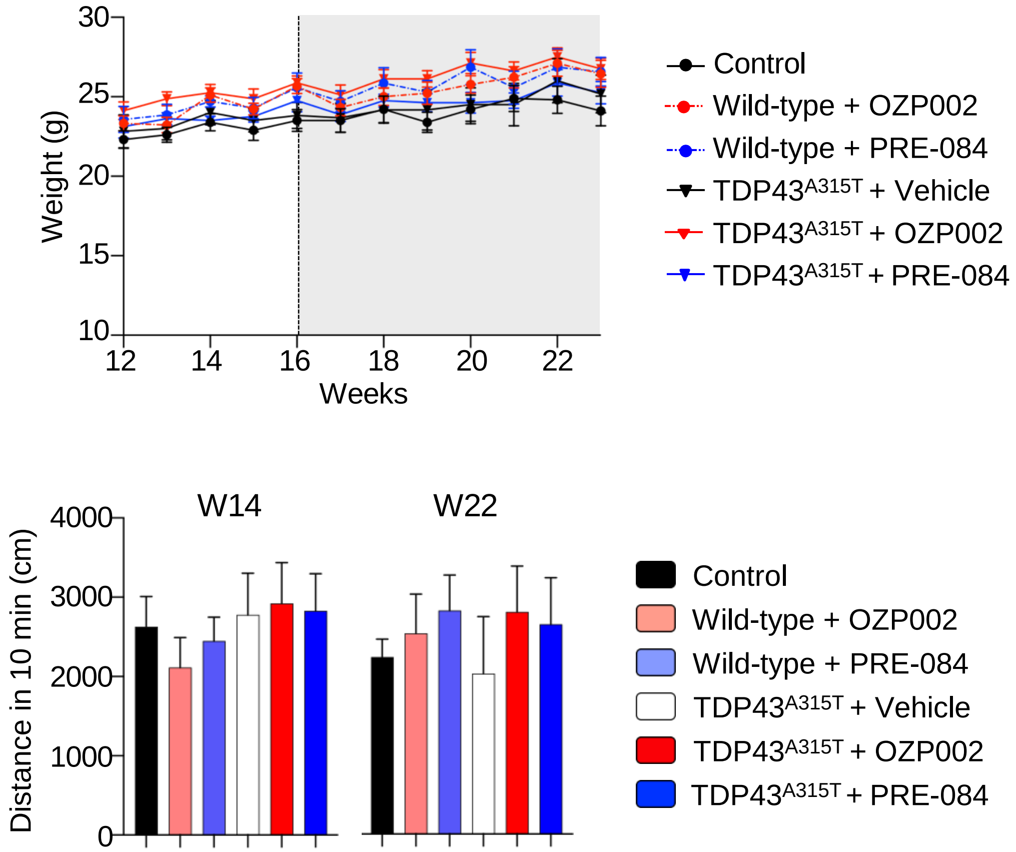

Figure S5. PRE-084 and OZP002 do not modify weight evolution and spontaneous locomotor activity. a Weight evolution of either wild-type mice or TDP43^{A315T} transgenic mice treated with saline solution, OZP002 (0.7 mg/kg), PRE-084 (0.3 mg/kg). **b** Spontaneous locomotor activity during open-field test obtained at 14 and 22 weeks of age on either wild-type mice or TDP43^{A315T} transgenic mice treated with saline solution, OZP002 (0.7 mg/kg), PRE-084 (0.3 mg/kg).

Table S1: Primers used for PCR and RT-qPCR

	Forward primer	Reverse primer
ef1α	5’-TTCTGTTACCTGGCAAAGGG-3’	5’-TTCAGTTTGTCCAACACCCA-3’
gclc	5’-ATCCGCCATAGGAGGGGTGA-3’	5’-TAGATGTGATCCGGCTTGGCA-3’
gclm	5’-CTGAAGCTTCACACGGGGAAT-3’	5’-CGCTCAATGTGCCTTGAATGC-3’
pgd	5’-ATGCAGCTGATCTGTGAGGC-3’	5’-AGGAAGGAGTCCAGCTCTGTC-3’
prdx6	5’-GAAACAGTCATGCCTGGGATA-3’	5’-GACCAACTACAAACACGCAG-3’
hmox1a	5’-GGGCAGGACTTGGAGCACTT-3’	5’-GGACTGCTCTTGCCAATCTCT-3’
hmox2a	5’-ATGGCGGTCAGTGGAAACACAACC-3’	5’-GGCAACAGCAGCAACCAATGTGGC-3’

Table S2: List of antibodies

Antibodies	Dilution ratio<br>IP: Immunoprecipitation<br>I: Immunostaining	Companies	Product code
Rabbit anti-GFP	IP: 1/5000	Proteintech®, Rosemont, USA	50430-2-AP
Mouse anti-GFP	IP: 1/5000	Proteintech®, Rosemont, USA	66002-1-1g
Rabbit normal IgG	NA	Invitrogen, Walthan, USA	10500C
Mouse normal IgG	NA	Santa Cruz Biotechnology, Dallas, USA	SC-2025
Mouse anti-BiP	IP: 1/500	BD Biosciences, Franklin Lakes, USA	610979
Rabbit anti-BiP	IP: 1/1000	Proteintech®, Rosemont, USA	11587-1-AP
Mouse anti-NRF2	IP: 1/1000	MBL Life Science, Carlsbad, USA	M200-3
Mouse anti-S1R	IP: 1/1000	Santa Cruz Biotechnology, Dallas, USA	B5
Rabbit anti-S1R	IP: 1/500	Proteintech®, Rosemont, USA	15168-1-AP
Rabbit anti-Keap1	IP: 1/2000	Proteintech®, Rosemont, USA	10503-2-AP
Mouse anti-NeuN	I: 1/400	Merck, Saint-Quentin-Fallavier, France	MAB377
Chicken anti-GFAP	I: 1/400	ABCAM, Cambridge, UK	ab4674
Rabbit anti-IBA1	I: 1/500	ABCAM, Cambridge, UK	ab5076
Rabbit anti-VGLUT1	I: 1/1000	Kind gift from Dr. Salah El Mestikawy	NA
Cy3 anti-mouse	I: 1/1000	Jackson ImmunoResearch, West Grove, USA	115-165-166
Alexa Fluor 488 Goat anti-rabbit IgG	I: 1/1000	Invitrogen, Walthan, USA	A-11008
Alexa Fluor 488 Goat anti-chicken IgG	I: 1/1000	ABCAM, Cambridge, UK	ab150169
Cy3 anti-rabbit	I: 1/1000	Jackson ImmunoResearch, West Grove, USA	111-165-003
