# Supplementary material for: Positive modulation of sigma-1 receptor: a new weapon to mitigate disease progression in amyotrophic lateral sclerosis
Source: Transl Neurodegener. 2025 Dec 15;14:68. doi: 10.1186/s40035-025-00527-z (PMC12703938; doi:10.1186/s40035-025-00527-z)

Original western blots for Figure 2e

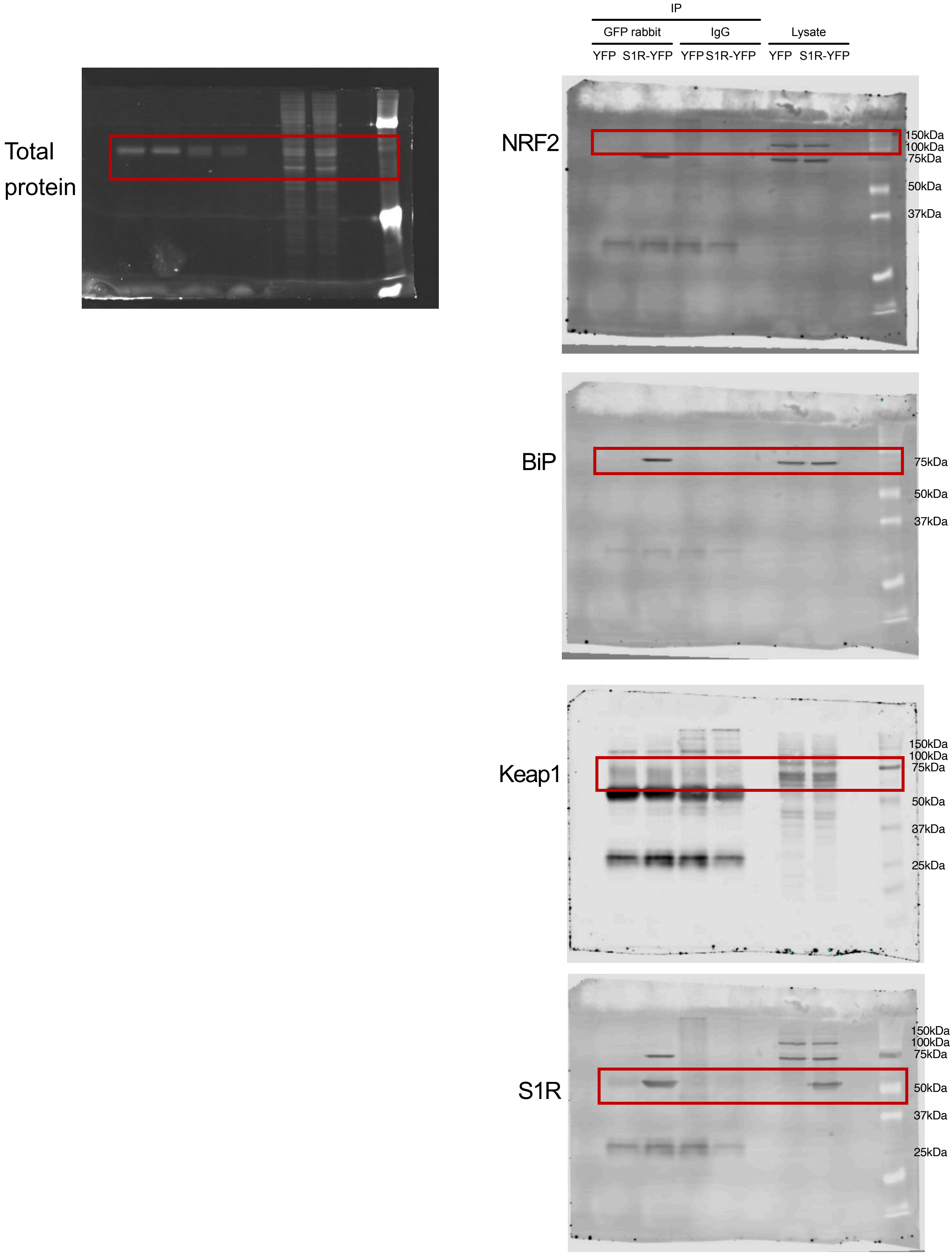

Original western blots for Supplementary Figure S4b

Total protein

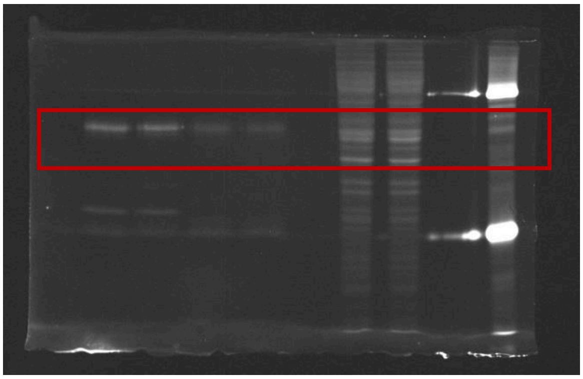

NRF2

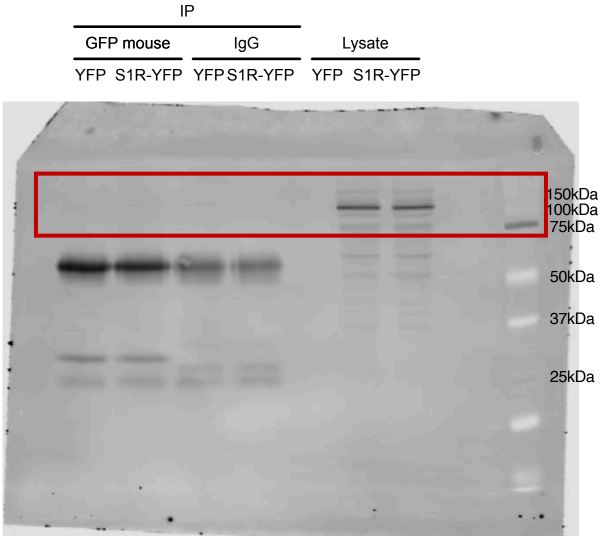

BiP

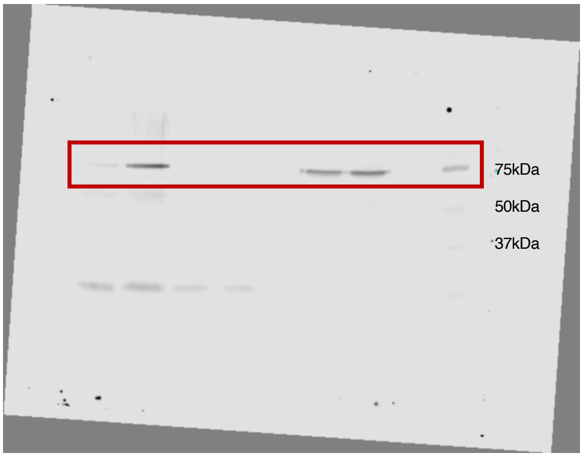

S1R

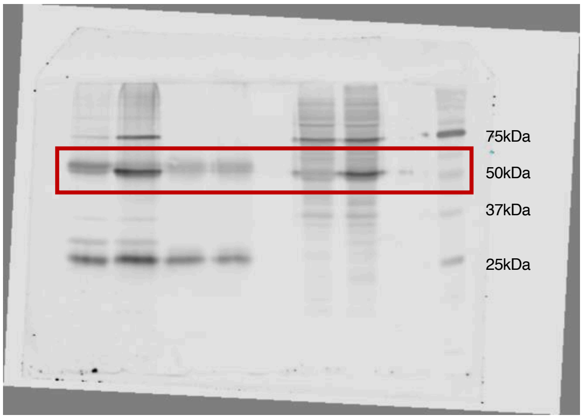

Supplement: Supplementary file 3 — Additional file 3 Uncropped western blots for Figures 2e and S4b. [file 40035_2025_527_MOESM3_ESM.pdf]
